# Effect of AKT inhibition on epithelial-mesenchymal transition and ZEB1-potentiated radiotherapy in nasopharyngeal carcinoma

**DOI:** 10.3892/ol.2013.1552

**Published:** 2013-08-29

**Authors:** WEIGUO CHEN, SIPEI WU, GONG ZHANG, WENJUN WANG, YONGSHENG SHI

**Affiliations:** 1 Department of Liver Disease Medicine, Pingxiang Hospital, Pingxiang, Jiangxi 337000, P.R. China; 2 Cancer Research Institute, Southern Medical University, Guangzhou, Guangdong 510515, P.R. China; 3 Department of Radiotherapy, Shanxi People’s Hospital, Taiyuan, Shanxi 030012, P.R. China; 4 Department of Anesthesiology, Nanfang Hospital, Southern Medical University, Guangzhou, Guangdong 510515, P.R. China

**Keywords:** AKT kinase inhibitor, nasopharyngeal carcinoma, epithelial-mesenchymal transition, AKT/ZEB1 pathway, radiotherapy

## Abstract

Radiotherapy is a major treatment regime for nasopharyngeal carcinoma (NPC), and although initial responses to a complete course of radiation are good, recurrence and metastasis are frequent events. A number of previous studies have observed that ionizing radiation (IR) may enhance the migratory and invasive properties of cancer cells through epithelial-mesenchymal transition (EMT). In the present study, a tumor cohort of 22 NPC and 7 normal cases (chronic inflammation only) were investigated and the expression of AKT was demonstrated to positively correlate with the expression of ZEB1. Following treatment with IR, 7/10 patients suffered recurrence and metastasis, in addition to high expression levels of phosphorylated AKT (S473) and ZEB1. The AKT inhibitor, GSK690693, inhibited AKT, blocked the expression of ZEB1 and vimentin and restored the expression of E-cadherin following IR, thus preventing the migration and EMT of the tumor cells. In addition, the inhibition of AKT via GSK690693 was shown to markedly increase the sensitivity of tumor cells to IR *in vitro* and *in vivo*. These observations indicate that GSK690693 may aid in the prevention of recurrence and metastasis following IR therapy in NPC patients.

## Introduction

Nasopharyngeal carcinoma (NPC) is a relatively uncommon type of malignant tumor that is distinct from other types of head and neck cancer with regard to epidemiology, method of treatment and prognosis (1,2). NPC has been identified as particularly prevalent in Southeast Asian populations (3). The anatomical location of NPC limits surgery to biopsies only (4) and the primary choice of treatment is radiotherapy (RT). However, conventional 2-dimensional RT has been shown to correlate with high rates of local recurrence and metastasis, particularly in patients with locally advanced NPC (5,6). Although radiotherapy is the cornerstone treatment for NPC, adjunctive chemotherapy has shown promise for improving tumor control and, possibly, the survival rate in cases of advanced NPC (7). The expression of E-cadherin and vimentin has also been demonstrated to correlate with metastasis formation in head and neck squamous cell carcinoma patients (8). Therefore, it is necessary to elucidate the effects of radiation on metastasis and recurrence and identify the relevant molecular mechanisms involved. An improved understanding of the mechanisms underlying the metastasis and recurrence of cancer following IR is likely to highlight and improve the therapeutic potential of radiotherapy for these types of cancer.

Epithelial-mesenchymal transition (EMT) is important for tumor progression and metastasis (9,10). Previous results have also demonstrated that ionizing radiation (IR) may enhance the invasiveness of cancer cells by inducing EMT (11). The loss of E-Cadherin expression has been shown to correlate with EMT and promote the radioresistance of human tumor cells (12). In addition, the activation of AKT has been demonstrated to correlate with the metastasis of NPC (13). In clinical practice, a marked correlation has been identified between the expression of active AKT and the outcome of treatment for breast cancer (14). Previous studies have also shown the importance of AKT inhibition for enhancing the efficacy of radiotherapy in head and neck cancer (15).

Since the activation of AKT is a critical element for cancer metastasis, recurrence and drug/radio-resistance, targeting the AKT pathway is a rational approach for cancer therapy. To understand the role of AKT in EMT and its association with radioresistance in NPC, a pan-AKT kinase inhibitor currently in clinical development for patients with various malignancies (16) was used to prevent the activation of AKT following radiation. GSK690693 is a novel ATP-competitive inhibitor of AKT that inhibits proliferation and induces apoptosis in a subset of tumor cells with a potency consistent with the intracellular inhibition of AKT kinase activity *in vitro* and *in vivo*(16–18). The results of the present study demonstrate that GSK690693 may block the phosphorylation of AKT and lead to the inhibition of cell metastasis and radioresistance via the regulation of ZEB1/E-cadherin levels in NPC cell lines.

## Materials and methods

### Cell culture and reagents

The NPC cell lines, CNE1 and CNE2, were purchased from American Type Culture Collection (ATCC, Manassas, VA, USA) and were maintained in RPMI-1640 medium supplemented with 10% newborn calf serum. The cells were maintained at 37°C in a humidified atmosphere of 5% CO_2_ and 95% air as recommended. For the EMT models, the cells were seeded and grown for 72 h, followed by being exposed to 5 Gy IR. The AKT inhibitor, GSK690693, was obtained from Selleck Chemicals (Houston, TX, USA).

### Western blotting

Whole cell extracts were prepared by washing the cells in cold PBS and lysis in a buffer containing 50 mM Tris-HCl (pH 7.5), 150 mM NaCl, 1% Nonidet P-40, 0.25% sodium deoxycholate, 1 mM Na_3_VO_4_ and 1 mM NaF, in addition to complete protease inhibitor cocktail tablets (Roche Diagnostics GmbH, Mannheim, Germany). Protein concentrations were determined using bicinchoninic protein assay reagents according to the manufacturer’s instructions (Pierce Biotechnology, Inc., Rock-ford, IL, USA). Antibodies against ZEB1, E-cadherin, vimentin, snail, GAPDH (1:500; Santa Cruz Biotechnology, Santa Cruz, CA, USA), AKT and phospho-AKT (1:1,000; Ser473; Cell Signaling Technology, Danvers, MA, USA) were used.

### Cell invasion assay

Matrigel invasion chambers (BD Biosciences, Franklin Lakes, NJ, USA) were rehydrated in DMEM for 2 h and then placed in 0.75 ml DMEM supplemented with 5% fetal calf serum. Following 72 h of IR or drug treatment for 48 h, 1.5×10^4^ cells suspended in 0.5 ml DMEM were seeded onto Matrigel chambers and allowed to invade for 12 or 24 h. The cells on the upper surface were gently removed with a cotton bud and the cells that had migrated through the 8-mm pores were fixed with 4% paraformaldehyde for 15 min and stained with 0.1% crystal violet for 10 min. The membranes were washed, removed and mounted onto a glass slide, and the level of invasion was quantified by visual counting using a microscope (magnification, ×20).

### Immunofluorescence

The cells were plated onto glass cover slips in 6-well culture plates, then fixed with 4% paraformaldehyde for 10 min, permeabilized with 0.2% Triton X-100 for 5 min and blocked with 1% BSA in PBS for 30 min. E-cadherin and vimentin (1:100) and AKT and phospho-AKT (p-AKT; Thr308; 1:200) were applied to sections overnight at 4°C. Nuclei were visualized by staining with DAPI (Sigma-Aldrich, St Louis, MO, USA) and images were captured on an inverted phase/fluorescence microscope (Nikon Eclipse 80i; Nikon, Amsterdam, Netherlands).

### Xenograft experiments

Animal studies were conducted in strict accordance with the principles and procedures approved by the Committee on the Ethics of Animal Experiments of Southern Medical University (Guangzhou, Guangdong, China). Nude mice (BALB/C nu/nu) were provided with autoclaved water and laboratory rodent chow. A volume of 100 μl culture medium mixed with Matrigel (BD Biosciences) containing 3×10^6^ CNE2 cells was transplanted into the flanks of the mice by subcutaneous injection. The tumor volume was monitored every 5 days and calculated as follows: Volume (mm^3^) = (a × b^2^) / 2, where a indicates the largest diameter and b the perpendicular diameter. Once tumors had reached ~70 mm^3^, the mice were randomly distributed into four groups (three mice/group) and treated with 30 mg/kg GSK690693 AKT inhibitor for 3 weeks (weekend rest) or 3×5 Gy IR. The tumor volume was monitored at various times for up to 24 days.

### Patient and primary NPC samples

Established protocols were followed with regard to written informed consent and anonymity. Tissues were obtained retrospectively from 22 NPC and 7 normal patients (chronic inflammation only) under strict anonymity from historical collections in the Department of Pathology, The First Affiliated Hospital of Zhengzhou University (Zhengzhou, China). All samples were fresh-frozen in liquid nitrogen following surgery and stored at −80°C. Frozen tissue samples were homogenized using the Tissue Ruptor (Qiagen, Hilden, Germany) prior to RNA extraction. Total RNA was extracted using TRI Reagent (MRC, Inc., Cincinnati, OH, USA) according to the manufacturer’s instructions. To determine the AKT and ZEB1 status of the primary NPC samples, PCR products of AKT and ZEB1 were used from genomic DNA. Written informed consent was obtained from each patient and the study was approved by the Ethics Committee of Nanfang Hospital (Guangzhou, China).

### Side population (SP) cell analysis by flow cytometry

The cells were analyzed by FACS when they had reached the alogarithmic growth phase (24 h after replating). The cells were digested with 0.25% trypsin (Giboc, Carlsbad, CA, USA), washed twice with calcium/magnesium-free PBS, re-suspended in ice-cold RPMI-1640 culture (supplemented with 2.5% FBS) at a concentration of 1×10^6^ cells/ml and incubated at 37°C in a 5% CO_2_ incubator for 10 min. The DNA binding dye, Hoechst 33342 (Sigma-Aldrich), was then added at a final concentration of 5 mg/ml and the samples were incubated for 90 min in the dark with periodic mixing. The cells were washed twice with PBS, then 1 mg/ml propidium iodide (Sigma-Aldrich) was added and the cells were kept at 4°C in the dark prior to analysis by a FACS Aria Flow Cytometer (Becton-Dickinson, Franklin Lakes, NJ, USA). Since Hoechst 33342 is extruded from cells treated with verapamil (a calcium ion channel antagonist)-sensitive ABC transporters, a subset of the cells were incubated with 50 mmol/l verapamil for 30 min at 37°C prior to the addition of Hoechst 33342.

### Colony formation assays

The cells were counted, plated in triplicate at 200 cells per well in six-well plates and cultured with RPMI-1640 complete culture for 12 h. Next, the cells were treated with 5 Gy irradiation or the addition of 10 μM AKT inhibitor (GSK690693) into the growth media at the indicated concentrations and allowed to form colonies for 16 days. Once the majority of the cell colonies had expanded to >50 cells in the control group, they were washed twice with PBS, fixed in methanol for 10 min and stained with crystal violet for 10 min at room temperature. Subsequent to washing out the dye, the colonies that contained >50 cells were counted and the results were compared. The colony-forming efficiency (CFE) was the ratio of the colony number to the transplanted cell number.

### Tumorsphere formation assay

Single-cell suspensions of cell lines or cells isolated from pleural effusions were suspended at a density of 4×10^4^ cells/ml in Dulbecco’s modified Eagle’s medium/F-12 containing 5 mg/ml insulin, 0.5 mg/ml hydrocortisone, 2% B27 (Invitrogen Ltd., Paisley, Scotland) and 20 ng/ml epidermal growth factor, and seeded into six-well plates (2.5 ml per plate) or T80 tissue culture flasks (10 ml per flask) coated with 1.2% polyhema. Cultures were fed weekly and passaged every 2 weeks. Tumorspheres were measured using Zeiss Axiovision software (Carl Zeiss, Jena, Germany). When passaged, the tumorspheres were harvested, incubated with trypsin for 3 min at 37°C and dispersed by pipetting with a 23-gauge needle. Subsequent to checking for single cells, the cells were pelleted and suspended in tumorsphere culture medium at 4×10^4^ cells/ml prior to replating in non-adherent plates or flasks.

### shRNAs

The pLKO.1 lentiviral shRNA vector and control shRNA targeting against GFP were purchased from Sigma-Aldrich. The Zeb1 targeting sequence in the shZeb1 plasmid was 5′-GGUUACGAACUAAGCUAUA-3′. The sense and antisense oligonucleotides were annealed and ligated into the pLKO.1 lentiviral vector.

### Statistical analysis

All experiments were repeated at least three times. Data are presented as the mean ± SD and P<0.05 was considered to indicate a statistically significant difference. All statistical analyses were performed by the v5.0 GraphPad Prism Program (GraphPad Software Inc., San Diego, CA, USA).

## Results

### IR-induced morphological changes and increased invasion of cancer cells are consistent with AKT activation in NPC

**Figure 1:**
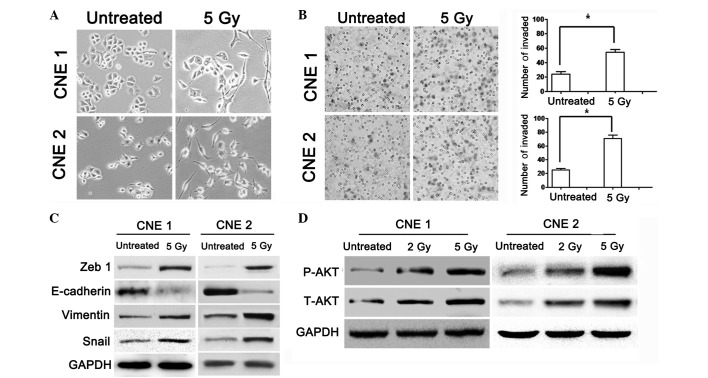
AKT suppresses EMT phenotype and stem cell properties. (A) Morphology of CNE1 and CNE2 cells 3 days after treatment with 5 Gy IR. (B) Representative immunohistochemical staining of pAKT and AKT and immunofluorescence using confocal microscopy. The differential expression of pAKT and AKT at the protein level in the CNE1 and CNE2 cells is shown. The cells were seeded in Matrigel-coated transwell inserts ands irradiated with two doses of IR (0 and 5 Gy). After 18 h, images of the cells that had invaded the membranes were captured under a light microscope and counted. The difference between the two groups was determined by Student’s t-test, as shown in the graphs. (C) Expression of ZEB1, vimentin, E-cadherin, snail and GAPDH was detected by western blot analysis in the untreated and irradiated CNE1 and CNE2 cells. (D) The CNE1 and CNE2 cells were cultured following irradiation with various doses (0, 2 and 5 Gy) and maintained in RPMI-1640 medium supplemented with 10% newborn calf serum for 72 h. Total lysates were run on SDS-PAGE, blotted and stained with antibodies against pAkt and Akt. Anti-GAPDH served as an internal control. ^*^P<0.05 vs. untreated (magnification, ×1,000; scale bar, 20 μm). EMT, epithelial-mesenchymal transition.

IR induces morphological changes and EMT in various types of carcinoma (19–21). To verify the changes in NPC cells following exposure to IR, the NPC cell lines, CNE1 and CNE2, were irradiated with 5 Gy shortly following attachment. The morphology of the irradiated cells was spindle-shaped and elongated when compared with that of the control group (Fig. 1A). To investigate whether IR may promote the migration and invasiveness of carcinoma cells, cell invasion assays using untreated and irradiated NPC cells were conducted. Irradiated cells exhibited increased motility and invasiveness (Fig. 1B). Western blot analysis of the irradiated group revealed the increased expression of vimentin, snail and ZEB1 and the downregulation of E-cadherin (Fig. 1C). To identify whether the phosphorylation of AKT is involved in IR-induced cell invasion, the expression of AKT and p-AKT (Ser473) was primarily examined in the NPC cells treated with various doses of IR. Total AKT and p-AKT protein expression levels in the IR-treated and untreated groups were determined by western blot analysis (Fig. 1D).

### Correlation between the expression of AKT and ZEB1 in human primary and metastatic NPC samples

**Figure 2:**
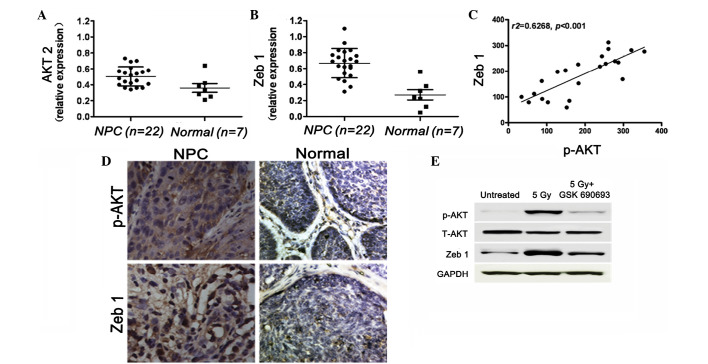
Expression of (A) AKT and (B) ZEB1 mRNA in 22 NPC patient and 7 normal samples. (C) Scatterplot demonstrating the correlation between ZEB1 and AKT. (D) IHC for pAKT, AKT and ZEB1 expression in human NPC patient and normal samples (E) Western blot analysis for total protein extracts of pAKT, AKT and ZEB1 from NPC cells treated with 5 Gy IR and cotreated with IR and GSK690693. GAPDH was used to determine equal protein loading. Normal, chronic inflammation of nasopharynx or tissues adjacent to NPC; NPC, nasopharyngeal carcinoma; IHC, immunohistochemistry; IR, ionizing radiation.

Notably, previous studies have demonstrated that the phosphorylation of AKT correlates with poor prognosis and radioresistance (22). The current study examined the expression of AKT and ZEB1 using qPCR in 22 NPC cases and 7 normal cases (chronic inflammation only) as controls. The results showed that the expression of AKT was higher in the NPC group when compared with that of the control group. The expression pattern for ZEB1 was similar to that of AKT in the 22 NPC and 7 normal cases (Fig. 2A and B). The correlation between p-AKT (Ser473) and ZEB1 expression was determined using immunohistochemistry and a marked correlation was identified (r^2^=0.6268; P<0.05; Fig. 2C and D). Based on these results, it may be hypothesized that patients with high levels of p-AKT and ZEB1 expression are at a higher risk of metastasis. GSK690693 is selective for AKT isoforms compared with the majority of other kinase families and has been shown to reduce the levels of p-AKT substrates *in vivo*(16,23). The results of the present study showed that IR induces AKT phosphorylation and the expression of ZEB1 in NPC cells. The NPC cells cotreated with shZEB1 and IR showed no change in expression when compared with the AKT and IR groups. Next, the GSK690693 AKT inhibitor was applied to the NPC cells, which were then treated with 5 Gy radiation (Fig. 2E).

### GSK690693 AKT inhibitor blocks IR-induced EMT and stemness in NPC

**Figure 3:**
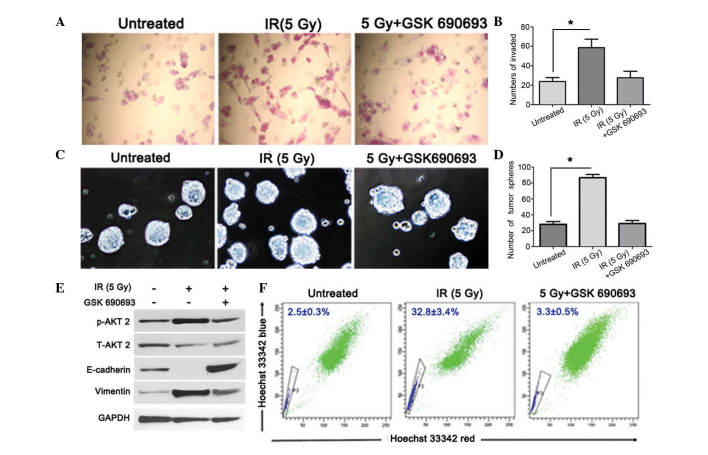
(A) Migration assays of CNE2 cells from parental (non-irradiated), 5 Gy IR and IR- and GSK690693-cotreated cells. (B) Summary graphs for migration and invasion from crystal violet stained cell counts of >10 representative fields. (C) Tumor sphere formation in CNE2 cells following no treatment, 5 Gy IR treatment and cotreatment with IR and GSK690693. (D) Spheres in each group (n=3) were counted in 10 fields. (E) Immunoblot analysis of pAKT, AKT, E-cadherin and vimentin protein levels in total protein extracts from CNE2 cells that were untreated, 5 Gy IR-treated or cotreated with IR and GSK690693. GAPDH was used to determine equal protein loading. (F) CNE2 cell lines were subjected to 5 Gy IR and cotreatment with IR and GSK690693, followed by Hoechet 33342 staining and flow cytometry analysis (scale bar, 200 μm). Data are presented as the mean ± SD. ^*^P<0.05 vs. untreated.

To clarify the efficacy of GSK690693 in the irradiated NPC cells, the invasion of the irradiated cells treated with the GSK690693 AKT inhibitor was compared with that of cells treated with IR only using a cell invasion assay. Cell invasion was identified to be higher in the irradiated NPC cells compared with the untreated cells (Fig. 3A and B), indicating that the AKT inhibitor may block IR-triggered cell invasion by inhibiting AKT phosphorylation. Side population (SP) cells and tumor sphere cells have stem cell characteristics that were hypothesized to be enriched of cancer stem cells in NPC (24). The rate of tumor sphere formation was ~24 spheres/1,000 cells in the untreated group and was identified to increase to ~83 spheres/1,000 cells following treatment with IR (Fig. 3C and D). To examine the correlation between AKT phosphorylation and the EMT activator (vimentin) and inhibitor (E-cadherin) in IR, the expression levels of these EMT-associated markers in IR-treated NPC cells were compared with that of cells cotreated with IR and GSK690693. IR-induced p-AKT activation in NPC cells was shown to correlate with a reduction in the levels of E-cadherin and an increase in vimentin expression. In the NPC cells cotreated with IR and GSK690693, the phosphorylation of AKT was inhibited compared with that of cells treated with IR only (Fig. 3E). In the present study, the treatment of irradiated NPC cells with GSK690693 reduced the percentage of SP cells (Fig. 3F).

### GSK69069 AKT inhibitor modulates radioresistance in NPC cancer cells in vivo

**Figure 4:**
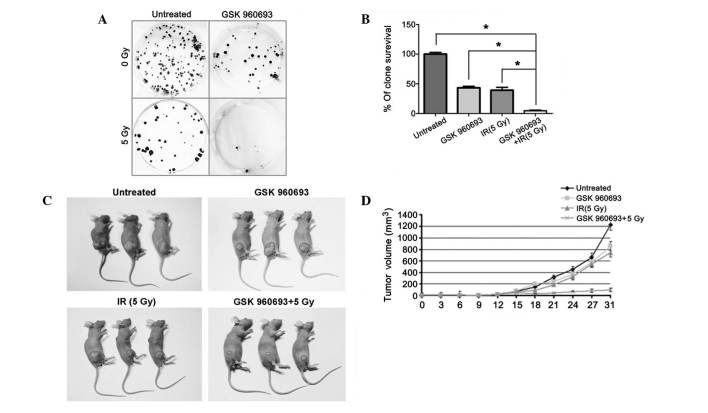
(A) Colony formation assay of CNE2 cells that were untreated, treated with 5 Gy IR or GSK690693 and cotreated with IR and GSK690693. (B) Colonies were counted in each well following the indicated treatment. (C and D) Tumor volume (mm^3^) in xenografts treated with saline, 5 Gy IR and GSK690693 or cotreated with IR and GSK690693 at days 3, 6, 9, 12, 15, 18, 21, 24, 27 and 31. Data are presented as the mean ± SD. P<0.05.

To examine the effects of the GSK690693 AKT inhibitor on NPC cells during radiotherapy *in vivo*, clone formation was compared in the untreated, GSK69069- or IR-treated and IR and GSK690693-cotreated NPC cell groups (Fig. 4A and B). Following treatment with GSK690693 and normalization against the control, ~62% of clones formed in the GSK690693 group and ~38% of clones were viable in the IR-treated group. However, following cotreatment with IR and GSK690693, >95% of the clones were killed. To determine the contribution of GSK690693 to the increased sensitivity of NPC cells to IR administration *in vivo*, the mice with tumors (70 mm^3^) that arose 10 days after injection were treated with 30 mg/kg GSK690693 every 3 days for 3 weeks (weekend rest), 3×5 Gy IR or combinations of GSK690693 with IR (Fig. 4C and D). As predicted, IR treatment caused significant regression of the tumor, but relapse of the disease occurred after 40 days. In the cells treated with GSK690693 only, an extremely small effect was noted on tumor growth and this was hypothesized to be due to the insensitivity of NPC to GSK690693 *in vivo*. Notably, cotreatment with GSK690693 and IR resulted in marked regression of tumor growth and relapse was prevented. Therefore, these results indicate that the GSK690693 AKT inhibitor potentiates radiotherapy *in vivo*.

## Discussion

Previous studies have reported that IR may enhance the metastatic potential of residual cancer (25). In addition, the activation of AKT in NPC cells during radiotherapy has been identified to correlate with cancer cell metastasis (13). The present study identified that the progeny of irradiated NPC cells are notably sensitized to undergo AKT phosphorylation-induced EMT. IR-induced AKT phosphorylation caused a phenotypic transition that occurred in the progeny of cells irradiated once and continued to persist during the activation of AKT. This resulted in increased motility, enhanced invasion, disrupted epithelial morphogenesis and an increased expression of mesenchymal markers.

AKT has previously been hypothesized to function as a positive and negative effector of mammary tumorigenesis, a tumor suppressor at early stages and a stimulator of tumor invasion at later stages (26). In addition, the phosphorylation of AKT has been demonstrated to correlate with a poor prognosis in a number of types of human cancer (27). According to the results of the present study, AKT exhibited an intimate correlation with ZEB1 and was upregulated in residual NPC cells following IR. Activation of ZEB1 by IR induced the mesenchymal markers, vimentin and snail, but the epithelial marker, E-cadherin, was suppressed. The aberrant expression of vimentin and snail in epithelial tumors has been hypothesized to promote the migration and invasion of carcinoma cells (28). In the present study, the expression of ZEB1 and AKT was compared and the correlation between p-AKT (Ser473) and ZEB1 was examined in clinical specimens. The levels of ZEB1 expression were demonstrated to correlate with p-AKT levels in NPC, however, GSK69069 prevented the activation of ZEB1 following IR. Supplementation of the NPC cells with low concentrations of GSK69069 was sufficient to prevent IR-induced EMT *in vitro*. The AKT inhibitor was used to knockdown the AKT gene in the NPC cells, and using immunohistochemical analyses of tumor xenografts previous studies have identified that repeated doses of GSK690693 reduce the number of pAKT substrates *in vivo*(16).

Consistent with the *in vitro* results, the expression of AKT and ZEB1 in the tissue from the untreated group increased following IR, but was shown to decrease in the groups treated with IR and GSK69069. IR led to the activation of mesenchymal markers, including vimentin and snail, and the suppressed expression of E-cadherin. It has been previously demonstrated that snail and slug are critical for cancer cells to acquire stem cell and EMT characteristics, including radioresistance and drug resistance (29). The loss of E-cadherin has been demonstrated to correlate with EMT and promote radioresistance in human tumor cells (12). Constitutively-activated AKT in BDC cells has also been shown to correlate with radioresistance (30) and AKT has been hypothesized to be important in the feedback loop whereby the IR-induced activation of AKT increases the radioresistance of GBM cells (31). Targeting the AKT signaling pathway may therefore have important therapeutic implications when combined with IR in the treatment of a subset of brain tumor patients. Increased AKT activation has been shown to correlate with radioresistance in various types of tumor and, in the present study, AKT activation was observed in residual cells following IR. Using NPC cells treated with the GSK69069 AKT inhibitor, the inhibition of IR-induced AKT activation was shown to increase radiosensitivity.

In conclusion, the observations of the current study have led to a number of hypotheses. Firstly, that IR-induced EMT activation of AKT occurs via the ZEB1 pathway and secondly, that activation of AKT is involved in radioresistance and EMT following IR in NPC. In addition, the novel AKT inhibitor, GSK69069, may block the AKT/ZEB1/E-cadherin/vimentin pathway, increase radiosensitivity and prevent recurrence and metastasis following IR therapy in NPC patients.
